# The mediation effects of moral resilience on the relationship between hospital ethical climate and work engagement among nurses: a cross-sectional study

**DOI:** 10.1186/s12912-025-04216-0

**Published:** 2025-12-11

**Authors:** Wenwen Zhang, Jing Zhang, Jing Wang, Qun Zhang, Xiaoyan Wu

**Affiliations:** 1 Rehabilitation Center, Qilu Hospital of Shandong University, Jinan, China; 2 Department of Nursing, Qilu Hospital of Shandong University, Nursing Theory & Practice Innovation Research Center of Shandong University, Jinan, China

**Keywords:** Moral resilience, Hospital ethical climate, Work engagement, Mediating effect

## Abstract

**Purpose:**

Nurses work in complex healthcare environments characterized by high-stakes decisions, moral dilemmas, and ethical challenges. While prior studies link hospital ethical climate to work engagement, the mechanisms underlying this relationship remain under explored. The purpose of this study To explore the mediation effects of moral resilience on the relationship between hospital ethical climate and work engagement among nurses.

**Methods:**

A total of 281 Chinese nurses completed an online survey in October 2024, including Hospital Ethical Climate Survey, Rushton Moral Resilience Scale, and Utrecht Work Engagement Scale. Pearson’s correlation was performed to examine the associations among the three variables. The mediation model was analyzed using SPSS 25.0 and the PROCESS macro 4.3.

**Results:**

Hospital ethical climate was positive associated with moral resilience(*r* = 0.491, *p* < 0.01) and work engagement(*r* = 0.625, *p* < 0.01). Moral resilience was positive associated with work engagement (*r* = 0.426, *p* < 0.01). In the mediation model, the direct effect of hospital ethical climate on work engagement was significant (*β* = 0.377, 95%CI = 0.306–0.449), and moral resilience significantly mediated this relationship (*β* = 0.053, 95%CI = 0.013–0.097). The mediation effect accounted for 12.33% of the total effect.

**Conclusion:**

The results revealed moral resilience played a mediating role in the relationship between hospital ethical climate and work engagement in clinical nurses. Nursing administrators should prioritize cultivating hospital ethical climate and enhancing moral resilience to improve clinical nurses’ work engagement.

## Background

With the continuous advancement of medical technology and the increasing workload, the mental health problems of nurses have significantly increased [1]. As frontline healthcare providers, clinical nurses often face substantial stress and overwork stemming from the high risks, heavy responsibilities, and intense emotional investment inherent in their roles—making them particularly vulnerable to mental health challenges such as anxiety, depression, job burnout, and compassion fatigue [2, 3]. These issues not only correlate with higher rates of chronic diseases and mental disorders among nurses but also adversely affect their interpersonal relationships and family life [4]. More importantly, they compromise patient safety and the quality of care delivered [5], and may even lead to the turnover of experienced nurses, seriously undermining the stability of the nursing workforce [6]. Addressing nurses’ mental health is therefore crucial to safeguarding their well-being and sustaining high-quality healthcare services. However, while the importance of improving nurses’ mental health is widely recognized, existing interventions often lack a solid theoretical foundation rooted in the ethical and psychological mechanisms underlying nurses’ work experiences. Specifically, research exploring how contextual factors (e.g., hospital ethical climate) and individual psychological resources (e.g., moral resilience) interact to influence nurses’ work engagement—an key indicator of their professional well-being and care quality—remains insufficient. To address this gap, this study investigates the relationships between the hospital ethical climate, moral resilience, and work engagement among Chinese nurses, with a specific focus on the mediating role of moral resilience. The findings are expected to provide new theoretical insights and practical guidance for designing ethical and psychological interventions to enhance nurses’ work engagement and improve their mental health.

Although the significance of improving nurses’ mental health for improving the quality of care has been fully recognized, comprehensive research on the relationship between moral resilience, hospital ethical climate and work engagement is still lacking. Therefore, the aims of this study were to explore the relationship between hospital ethical climate, moral resilience, and work engagement, and to explore the mediating role of moral resilience between hospital ethical climate and work engagement, providing new theoretical insights for improving the mental health of nurses. The mental health of nurses caused by ethical issues are often overlooked, among which moral distress is the prevalent and significant among these concerns [7]. In the United States, a study surveyed 2,073 clinicians from 20 states, covering areas such as primary care, dentistry, and behavioral health, indicating that moral dilemmas are widespread in primary care practices [8]. Moral distress refers to the distress nurses experience when institutional constraints make it nearly impossible to act according to their own moral judgment [9]. Moral distress may lead to burnout, emotional exhaustion, lack of empathy, numbness, feelings of disengagement, and job dissatisfaction, which may increase staff turnover, reduce quality of care, and make patient outcomes poor [10–12]. Although the moral distress experienced by nurses are usually regarded as negative, they are not entirely harmful. In fact, moral distress can prompt nurses to reflect on themselves, thereby promoting the development of innovative problem-solving strategies and improving the quality of care. Moral distress can serve as a catalyst for nurses to reflect on their own behaviors and inner selves. This kind of self-reflection can promote the development of innovative problem-solving strategies, thereby improving the quality of care. Ultimately, this process cultivates a higher level of moral resilience. Moral resilience refers to the capacity of an individual to sustain or restore their integrity in response to moral complexity, confusion, distress or setbacks [5], which was regarded as one of the effective strategies to deal with moral distress. Antonsdottir et al.‘s research conducted in five hospital systems in the eastern United States shows that moral resilience can reduce moral distress, lower turnover rates, and decrease job burnout [13]. What’s more, Rushton believed that moral resilience was a key ability for nurses to maintain, restore and enhance mental health when encountering moral dilemmas [14].

Since various resources available to individuals play a significant role in promoting physical and mental health, the resource factor has received increasing attention from the academic community [15]. Hospital ethical climate is regarded as an important external resource. It refers to nursing staff perceptions of how ethical issues are addressed in their specific work environment [16], which largely depends on the relationships between nurses and the hospital, managers, physicians, patients, and colleagues [17]. Hosseinpour et al.‘s study demonstrated that a positive ethical climate, particularly through the provision of adequate team support and a fair power structure, can mitigate various risk factors, thereby effectively reducing the frequency and intensity of moral distress experienced by nurses [18]. It has been shown to impact an individual’s moral distress [19], moral courage [20], moral sensitivity [21], job satisfaction [22], and intention to remain in their roles [23]. Besides, positive ethical climate was associated with increased moral resilience among head nurses [24]. Spilg et al.‘s study has found that higher moral resilience was significantly associated with greater support from one’s employer and co-workers [25]. A good organizational ethical atmosphere and ethical leadership style are indispensable for developing and shaping of nurses’ moral resilience [26]. A favorable ethical climate can reduce the conscience pressure of nurses [27], alleviate their sense of job alienation [28], stabilize their psychological state and promote their physical and mental health development.

Work engagement refers to a positive, fulfilling and work-related psychological state, characterized by vigour, dedication and absorption [29]. Previous studies have shown that work engagement plays a positive role in the physical and mental state and career development of nurses, which helps to improve nurses’ job satisfaction, performance, nursing quality, and ensure the safety of patients [30, 31]. Work engagement is a protective factor that can alleviate nurses’ psychological distress, job burnout and turnover rates [32, 33]. Therefore, an increasing number of hospital managers are exploring factors that can improve nurses’ work engagement. Liu et al.‘s study indicated that moral resilience was significantly positively correlated with work engagement [34]. According to a study by Galinis et al. [35], moral resilience can mitigate key adverse work conditions among nurses, including quiet resignation, job burnout, and the intention to leave the profession. By mitigating these factors, the research indirectly posits that moral resilience may foster positive work outcomes, such as greater job engagement. Moral resilience, as a positive psychological attribute demonstrated by individuals when facing moral dilemmas and challenges, enables individuals to adhere to their core values and moral principles while devoting themselves wholeheartedly to their work. According to Rushton [36], a nurse’s ability to handle moral adversity in clinical practice can be enhanced by cultivating moral resilience, such as moral confidence and moral ability. This improvement is conducive to establishing a healthy working environment, reducing the turnover rate of nurses, and ultimately increasing work engagement. The research review conducted by Borrelli et al. suggests that ethical climate plays an instrumental role in alleviating employees’ job burnout and improving work engagement [37]. Furthermore, ethical climate has been proven by Bakker et al. [38] to enhance work engagement and could be a useful factor in ensuring the mental health of workers. Since ethical climate is defined as the perception of moral values in the workplace, the role of morality in enhancing work engagement seems to be more relevant when shared among colleagues. According to the Job Demands-Resources (JD-R) model [39], work engagement is a motivational consequence of resources, which are categorized as either job resources (external) or personal resources (internal). These resources affect engagement through distinct stress or motivation pathways. Thus, a hospital ethical climate can be viewed as a job resource, and an individual’s moral resilience as a personal resource. Nevertheless, the JD-R model does not explicitly delineate how these two types of resources interact under conditions of ethical strain. The relationship between moral resilience, hospital ethical climate and work engagement, as well as the mechanisms underlying these variables, remains unclear within the nursing field. In China, nurses face multiple challenges, including staffing shortages, low professional recognition, inadequate compensation, and limited career advancement opportunities. Although the national “Healthy China 2030” initiative emphasizes the psychological well-being of nurses and the quality of nursing care, and has introduced measures such as salary increases and improved working conditions to enhance nurses’ physical and mental health and stabilize the workforce, ethical aspects of nursing practice have yet to receive sufficient attention. Therefore, this study aims to investigate the relationships among the hospital ethical climate, moral resilience, and work engagement among Chinese nurses, specifically examining the mediating role of moral resilience.

### Hypotheses

Based on previous literature, we propose the following hypotheses:

H1: Hospital ethical climate positively correlates with moral resilience.

H2: Moral resilience positively correlates with work engagement.

H3: Hospital ethical climate positively correlates with work engagement.

H4: Moral resilience mediates the relationship between hospital ethical climate and work engagement.

## Methods

### Participants

A descriptive, cross-sectional study among nurses was conducted in a comprehensive class A tertiary hospital located in Jinan, Shandong Province, China, using convenience sampling. The inclusion criteria were as follows: (1) registered nurse, (2) officially employed by the hospital, and (3) volunteering to participate in this study. The exclusion criteria were: (1) absence during the period of data collection, and (2) unwilling to participate in this study. Sample size was calculated using G*Power 3.1.9.4. With parameters set as statistical power of 0.95, significance level of 0.05, 9 predictors, and effect size of 0.15, the minimum required sample size was 166. After accounting for an expected 20% dropout rate, this size was adjusted to 208. A total of 281 patients were finally enrolled in the study.

### Data collection

Data were collected in October 2024 through an online survey (https://www.wjx.cn). After obtaining approval from the hospital’s nursing management department, we sent the QR code along with a brief introduction of the research to the nurses through the hospital’s official nursing communication channels - mainly the internal WeChat groups covering all clinical departments. Participants filled out the questionnaire by scanning the QR code or clicking the link in the WeChat groups. Each IP address could only submit the questionnaire once. Only all the items required to be filled in, could the questionnaire be submitted. Participants were assured that their participation was voluntary, and that they could withdraw at any time. Collected data kept confidential and anonymous, and only used for this study. If they have any questions during the filling process, they could contact the researcher by phone. The survey was open for data collection over a 2-week period. At the end of the recruitment period, a total of 281 completed questionnaires were retrieved.

### Measures

#### Demographic characteristics of the sample

Demographics were collected with a questionnaire, including gender, age, marital status, education level, department, years of work experience, professional title, position, employment type, monthly income, night shift frequency, and ethical training experience.

#### Rushton moral resilience scale

The Rushton Moral Resilience Scale (RMRS) [14] is designed to measure levels of moral resilience. This 17-item scale is arranged in 4 sub-scales: responses to moral adversity, personal integrity, moral efficacy, and relational integrity. The response format is a Likert-type scale with four response options (1 = Disagree, 2 = Somewhat Disagree, 3 = Somewhat Agree, and 4 = Agree), with higher total scores indicating greater moral resilience. The RMRS demonstrated convergent validity with the Connor Davidson Resilience Scale-10. The overall reliability is 0.84 and internal reliability for each sub-scale ranges from 0.50 to 0.78. The Chinese version RMRS was translated by Yang Qingqing et al. [40] in Aug. 2022, according to the opinions of the original author and experts, subject feedback, research group discussion, and Chinese language and cultural environment. Finally, a 16-item Chinese version of RMRS was formed. Its overall reliability is 0.763 and content validity index was 0.901.

#### Hospital ethical climate survey

Hospital Ethical Climate Survey (HECS) was developed by Olson [17] in the United States in 1998 and translated and validated by Wang et al. [41] for Chinese in 2018, including 5 dimensions, namely the relationship with nurses, patients, doctors, managers and hospitals, with a total of 25 items. Likert 5-point scale was used, with 1 = “almost never true” and 5 = “almost always true”. The total score of each item is summed up, and the higher the score, the more positive the perception of the ethical climate. The Cronbach’s α coefficient was 0.91 for total scale, ranging from 0.68 to 0.92 for each dimension.

#### Utrecht work engagement scale

The Utrecht Work Engagement Scale (UWES-9) developed by Schaufeli et al. [42] was used to evaluate nurses’ work engagement. The scale consists of 9 items, includng three dimensions: vigor, dedication, and absorption. Each item adopts the Likert 7-level scoring method, with scores ranging from 0 to 6 from “never” to “always”, and the total score is 0 to 54. A higher score indicates a higher level of work engagement. The Chinese version of UWES-9 has been validated in previous studies on Chinese clinical nurses, showing satisfactory reliability and construct validity [43]. Its Cronbach’s α coefficient was 0.944 for the total scale.

### Statistical analysis

Data analysis was performed using SPSS 25.0 and the PROCESS macro 4.3. Means and standard deviations (SD) were used to describe the distributions of continuous variables, and frequencies and percentages were used to characterize categorical variables. Pearson’s correlation was performed to examine the associations among the three variables: moral resilience, hospital ethical climate and and work engagement. PROCESS Model 4 was adopted to investigate the mediating role of moral resilience between hospital ethical climate and work engagement among nurses. The statistically significance was defined as *p* < 0.05, and all tests were two tailed.

### Ethical considerations

This study was conducted in accordance with the Declaration of Helsinki and its subsequent amendments. Ethical approval for this study was obtained from the Research Ethics Committee of Qilu Hospital of Shandong University (No. KYLL-202409-026). Informed consent was obtained from all the subjects. All participants were assured that their participation was voluntary and anonymous.

## Results

### Characteristics of participants

Table 1 shows the demographics characteristics of the nurses. There were 281 participants, with a mean age of (33.7 ± 6.2), ranging from 20 to 54 years, and 260 (92.5%) of them were female. The average years of working was about (11.1 ± 7.1) years. 221 (78.7%) participants were married, and those with a bachelor’s degree accounted for the largest number at 235 (83.6%). Regarding nurses’ title, nearly half of the nurses (46.2%) were nurse-in-charge, and more than half of the participants (55.5%) had received training in medical ethics.

**Table 1:** Characteristics of the participants

Variables		Mean (SD)	*n*(%)
Age(years)		33.7 (6.2)	
Years of working		11.1 (7.1)	
Gender	Male		21 (7.5)
	Female		260 (92.5)
Marital status	Married		221 (78.7)
	Single		56 (19.9)
	Divorced or widowed		4 (1.4)
Education level	Junior college		2 (0.7)
	Bachelor degree		235 (83.6)
	Master or above		44 (15.7)
Department	Medical department		49 (17.4)
	Department of surgery		118 (42.0)
	Department of gynecology		16 (5.7)
	Department of pediatrics		28 (10.0)
	ICU		25 (8.9)
	Department of emergency		25 (8.9)
	Others		20 (7.1)
Position	Staff nurse		268 (95.4)
	Head nurse		13 (4.6)
Professional title	Nurse		28 (10.0)
	Senior nurse		118 (42.0)
	Nurse-in-charge		130 (46.2)
	Associate chief nurse		5 (1.8)
Monthly income (yuan)	≤ 5000		13(4.6)
	5001–10,000		94(33.5)
	10,001–15,000		144(51.2)
	>15,000		30(10.7)
Monthly number of night shift	0		59(21.0)
	1–5		75(26.7)
	6–10		99(35.2)
	>10		48(17.1)
Employment type	Contract		234 (83.3)
	Personnel agency		27 (9.6)
	Formal		20 (7.1)
Ethical training	Yes		156 (55.5)
	No		125 (44.5)

### Questionnaire scores

Descriptive results for the study variables are shown in Table 2. The mean scores of moral resilience, hospital ethical climate and work engagement were (2.77 ± 0.41), (4.19 ± 0.57) and (4.26 ± 1.08), respectively. The sub-dimensions’ total mean, standard deviation, and range were presented in Table 2.

**Table 2:** Descriptive results for moral resilience, hospital ethical climate and work engagement (*N* = 281)

Variables	Number of items	Range	Scores (Mean ± SD)	Means (Mean ± SD)
Moral resilience	16	29–61	44.33 ± 6.51	2.77 ± 0.41
Response to moral adversity	4	4–16	10.83 ± 2.83	2.71 ± 0.71
Relational integrity	5	5–20	13.05 ± 3.06	2.61 ± 0.61
Personal integrity	3	3–12	7.57 ± 1.25	2.52 ± 0.42
Moral efficacy	4	7–16	12.87 ± 1.88	3.22 ± 0.47
Hospital ethical climate	25	49–125	104.70 ± 14.13	4.19 ± 0.57
Relationship with nurses	4	7–20	17.04 ± 2.33	4.26 ± 0.58
Relationship with patients	4	8–20	16.89 ± 2.21	4.22 ± 0.55
Relationship with doctors	5	10–25	20.24 ± 3.23	4.05 ± 0.65
Relationship with managers	6	12–30	25.79 ± 3.62	4.30 ± 0.60
Relationship with hospitals	6	12–30	24.74 ± 3.74	4.12 ± 0.62
Work engagement	9	3–54	38.31 ± 9.71	4.26 ± 1.08
Vigor	3	2–18	13.32 ± 3.19	4.44 ± 1.06
Dedication	3	0–18	12.64 ± 3.65	4.21 ± 1.22
Absorption	3	0–18	12.35 ± 3.43	4.12 ± 1.14

### Correlation analysis of research variables

The results of the Pearson’s coefficient correlations are shown in Table 3. According to the results, there was a significant positive correlation between the total scores on moral resilience and hospital ethical climate (*r* = 0.491, *p* < 0.01). Furthermore, a positive correlation was observed between hospital ethical climate and work engagement (*r* = 0.625, *p* < 0.01), as well as between moral resilience and work engagement (*r* = 0.426, *p* < 0.01).

**Table 3:** The relationships among moral resilience, hospital ethical climate and work engagement (*N* = 281)

Variables	Moral resilience	Hospital ethical climate	Work engagement
Moral resilience	1		
Hospital ethical climate	0.491**	1	
Work Engagement	0.426**	0.625**	1

**P ≤ 0.01

### Testing the hypothesised model and parameter estimates

**Fig. 1:**
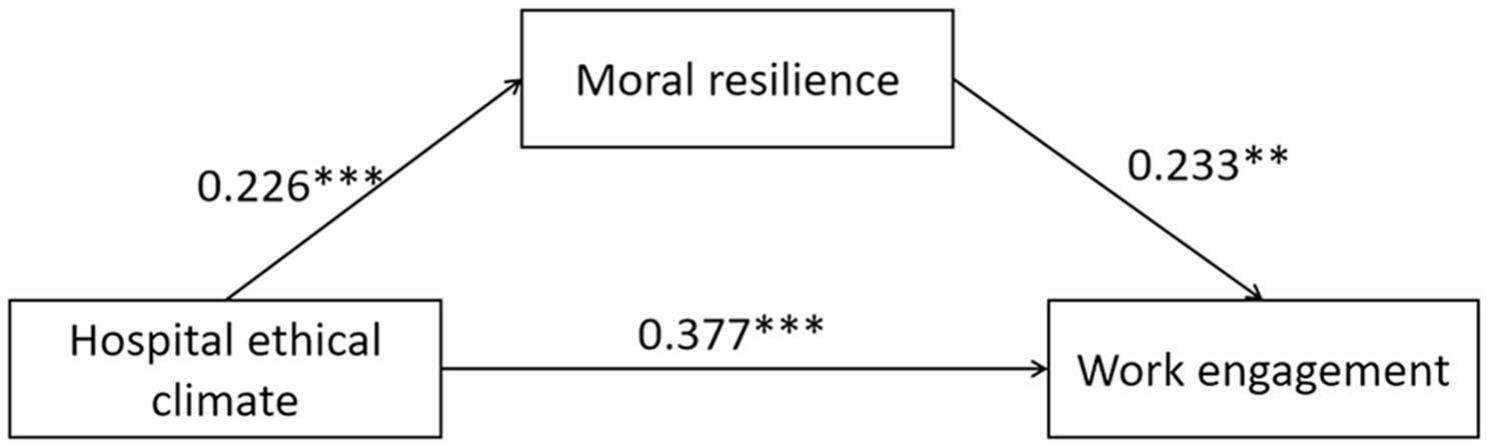
The mediation model. ***p < 0.001,**p < 0.01

We used the PROCESS macro 4.3 (Model 4) proposed by Hayes to examine the mediating effect of moral resilience on the relationship between hospital ethical climate and work engagement among nurses. Table 4 revealed that the 95% bootstrap confidence intervals for the direct effect of hospital ethical climate on work engagement, as well as the mediating effect of moral resilience, did not indude zero. In the mediation model, the direct effect of hospital ethical climate on work engagement was significant (*β* = 0.377, 95% CI = 0.306–0.449), and moral resilience significantly mediated this relationship (*β* = 0.053, 95% CI = 0.013–0.097). The mediation effect accounted for 12.33% of the total effect. The relationship between these variables is illustrated in Fig. 1, where the direct effect is 0.377 and the indirect effect is 0.053. Notably, the indirect effect accounts for 12.33% of the total effect, and the direct effect accounts for 87.67%.

**Table 4:** Total effect, indirect effect and direct effect

Effect	β	SE	95%CI	
			Lower	Upper
Direct effect	0.377***	0.036	0.306	0.449
Indirect effect	0.053***	0.022	0.013	0.097
Total effect	0.430***	0.032	0.367	0.493

***P ≤ 0.001

## Discussion

This study aimed to investigate the relationship between hospital ethical climate, moral resilience, and work engagement among clinical nurses while also examining the mediating role of moral resilience in this relationship. Our results revealed a significant positive correlation among hospital ethical climate, moral resilience, and work engagement. Importantly, moral resilience was identified as a mediator between hospital ethical climate and work engagement in clinical nurses. The findings significantly advance our understanding of how a positive ethical environment translates into greater nurse engagement, highlighting moral resilience as a crucial psychological mechanism.

Nurses in this study evaluated the hospital ethical climate positively and higher than neutral with a total sum of 104.70, which is consistent with previous studies conducted in Chinese nurses [44, 45]. Notably, this study contributes novel insights by verifying the ethical climate perception of nurses in Chinese tertiary hospitals against the backdrop of recent domestic healthcare reforms (e.g., the implementation of the “Nine Guidelines for Honest Practice of Medical Institution Staff” since 2021 and the advancement of the “Healthy China 2030” strategy). However, the average score of this questionnaire was higher than that reported by nurses from cancer care institutions in Greece and Cyprus in 2019 [46], which can be attributed to inherent differences in cultural contexts and healthcare systems. We also found that nurses perceived more positive relationships with managers and peers than with doctors, which was consistent with previous studies [41, 47]. This phenomenon is rooted in China’s traditional medical hierarchy, where doctors have long held a dominant position in clinical decision-making, leading to potential imbalances in inter-professional communication and collaboration. In the Chinese context, the impact of the ethical climate on nursing professionals is particularly pronounced. A positive ethical climate in Chinese hospitals can effectively reduce nurses’ moral distress—an issue that has been exacerbated by heavy workloads and high clinical pressure in China’s healthcare system—thereby improving clinical decision-making quality and reducing medication errors [48]. Conversely, a negative ethical climate is more likely to fuel turnover intention among nurses, potentially exacerbating the existing nursing shortage in China [49]. To optimize the ethical climate within Chinese hospitals, the following targeted strategies are recommended: (1) Hospitals should formulate and implement comprehensive ethical guidelines and policies, ensuring they are effectively disseminated to all nursing staff; (2) Develop localized ethics training programs that emphasize inter-professional collaboration, such as doctor-nurse communication skills tailored to the context of Chinese medical culture, as well as the practice of ethical integrity; (3) Establish designated channels for nurses to raise ethical concerns, thereby facilitating the timely resolution of clinical ethical issues through hospital ethics committees.

Consistent with earlier research on Chinese nurses [50], our study found that Chinese nurses exhibited lower moral resilience (2.77 ± 0.41) compared to their Greek [51] and American counterparts [14, 52]. To contextualize these cross-cultural differences, it is necessary to first clarify the unique cultural and healthcare system characteristics in China that may shape moral resilience. Culturally, China’s collectivist orientation emphasizes group cohesion and harmony, which guides individuals to prioritize collective interests over personal expression when confronting challenges [53]. This stands in contrast to the individualistic cultures prevalent in Western nations, where personal autonomy and self-advocacy are prioritized—differences that may lead to distinct coping strategies for moral dilemmas. Systemically, the ethical climate in Chinese hospitals, influenced by this collectivist culture, often emphasizes hierarchical collaboration and consensus-building, which could indirectly impact how nurses navigate ethical conflicts. In addition, from the perspective of each dimension, moral efficacy scored the highest, followed by the the response to moral adversity and the relational integrity, and personal integrity was the lowest. This indicates that nurses have strong confidence in their ability to recognize and respond to ethical challenges effectively. They also demonstrate moderate capacity to cope with moral adversity and maintain integrity in interpersonal interactions. However, compared to other dimensions, nurses’ ability to adhere to their own moral principles and avoid compromising personal values under workplace pressures shows relatively more room for improvement. Moral resilience, an endogenous moral quality, can serve as a vital internal resource for nurses facing ethically fraught situations, enabling them to deal with moral distress [54]. As medical models evolve and new technologies integrate into nursing practice, the ethical dilemmas nurses face in clinical settings grow increasingly complex [55]. Regarding countermeasures, current support for moral resilience building among Chinese clinical nurses remains limited, with few targeted initiatives widely implemented. The most widely implemented strategy involves the systematic reinforcement of ethical education and training. Hospital-based ethical training fosters a deeper comprehension and application of nursing professional ethics, while also enhancing nurses’ sense of responsibility and empathy toward patients. Furthermore, such training equips nurses with the ability to maintain psychological stability, make reasoned decisions in morally complex and high-pressure scenarios, and achieve constructive self-recovery in the face of occupational challenges [56]. Mindfulness and self-reflection exercises have been implemented in Western nursing settings [57]; however, their applicability and effectiveness in the Chinese context remain to be verified. Therefore, nursing managers in China should prioritize these contextually appropriate supportive interventions to enhance clinical nurses’ moral resilience.

Our findings showed that the mean work engagement was 4.26 (SD = 1.08), which was slightly lower than that of Wang et al. [30] and Cai et al. [58], but higher than that of Gao’s study [59]. These discrepancies may reflect regional variations in work demands, performance expectations, and organizational support within China. These regional variations are closely associated with the unique context of China’s healthcare system and uneven regional development. Specifically, nurses in more developed regions often operate within better-resourced healthcare systems and receive stronger institutional backing, whereas those in underserved areas may face higher workloads with limited support. These factors likely contributing to differences in engagement levels. Compared with foreign research, our work engagement was lower than other studies conducted among Vietnamese nurse [60] and American Nurses [61], and higher than Iranian nurse [62]. It indicates that in different periods and across different countries, there are differences in aspects such as nurses’ leadership, social status, working environment, and salary. Consequently, there are also certain variations in the level of nurses’ work engagement. Work engagement may play a decisive role in aspects such as productivity, prevention of occupational burnout, shortage of healthcare workers, and service quality, all of which could shape the future of healthcare [63]. Therefore, nursing managers can take corresponding measures to enhance their work vitality and concentration to increase their level of work engagement, thereby promoting the development of future healthcare.

Current findings show that hospital ethical climate has a positive impact on moral resilience (*r* = 0.491, *p* < 0.01), and moral resilience had a positive impact on work engagement (*r* = 0.426, *p* < 0.01). These results support hypotheses 1 and 2. Yu et al. [24] demonstrated a significant and positive correlation between hospital ethical climate and moral resilience among head nurses, which aligns with our study’s conclusion. Ethical climate is an important organizational factor in ethical issues. Organizational ethical climate and ethical leadership style are indispensable in the development and shaping of moral resilience. A good hospital ethical climate can enhance nurses’ professional values and satisfaction, reducing their confusion and distress when facing moral distress [64]. What’s more, a positive hospital ethical climate provides institutional support that reduces nurses’ moral distress and creates conditions for the development of moral resilience. According to a cross-sectional study conducted by Clark et al. [65] examining resilience, moral distress, and workplace engagement, higher adaptability in nurses was linked to increased workplace engagement. This finding bolsters our conclusion that moral resilience positively relates to work engagement. Furthermore, previous studies [36] have confirmed that nurses’ ability to cope with moral distress in clinical practice can be enhanced through the cultivation of elements related to moral resilience, which can help establish a healthy working environment, reduce the turnover rate of nurses, and ultimately increase their work engagement.

Our results show a significant and positive correlation between hospital ethical climate and work engagement (*r* = 0.625, *p* < 0.01). Our hypothesis 3 was validated. A previous study [38] has shown that ethical climate could improve work engagement and could be a useful factor in ensuring employees’ mental health. According to a systematic review [37], ethical climate has been reported be positively associated with work engagement, which in turn reduced burnout. An organization’s ethical climate plays a critical role in mitigating worker burnout and promoting work engagement. The synergy between workplace ethics, heightened engagement, and reduced burnout contributes significantly to lower turnover intentions. A positive hospital ethical climate fosters nurses’ sense of belonging and irreplaceability, deepening their job embeddedness, boosting intrinsic motivation, and ultimately increasing work engagement. The evaluation of hospital ethical climate should be implemented as a routine evaluation to ensure the wellbeing of nurses through the implementation of a fair ethical climate and ethical leadership in the hospital.

Our mediation analysis demonstrated that moral resilience significantly mediates the relationship between hospital ethical climate and work engagement, with a mediating effect ratio of 12.33%. These results support hypothesis 4, confirming that hospital ethical climate contributes to work engagement both directly and indirectly through cultivating moral resilience. The hospital ethical climate, as the environmental perception of individuals regarding the practical orientation of ethical issues, has a significant impact on the occupational health of nurses. A positive ethical climate serves as the cornerstone for nurses in addressing ethical challenges. It not only helps them resolve conflicts and reduce moral distress but, more importantly, fosters the growth of nurses’ moral sensitivity [66] and resilience. This enables nurses to identify ethical issues at an early stage, maintain professional integrity in complex situations, and muster the moral courage to overcome internal and external obstacles [67]. Consequently, they can effectively mitigate the impact of moral distress. Moral resilience serves as vital protection for healthcare workers. It prevents ethical dilemmas, lessens moral injury, and counters burnout by enhancing their ability to manage moral complexity and reduce ongoing stressor accumulation [14]. According to the JD-R model, moral resilience, as an individual resource component of work engagement, can help individuals adjust their psychological state to deal with newly emerging moral distress events, alleviate moral distress, and thereby enhance the level of work engagement. Our study indicates that a positive hospital ethical climate strengthens nurses’ moral resilience, subsequently boosting their work engagement. This aligns with established research on moral resilience’s influence on nursing outcomes. Furthermore, moral resilience acts as a moderating factor between ethical climate and engagement, robustly validating this linkage. In conclusion, a positive hospital ethical climate helps nurses effectively handle moral challenges and emotional stress in their work by fostering moral resilience, thereby enhancing their work engagement.

Several limitations warrant consideration. Firstly, the reliance on convenience sampling within a single tertiary hospital limits the generalizability of findings. Expanding future research to include diverse geographic locations, hospital settings, and larger samples is recommended. Secondly, in our model, the characteristics of the participants were not included as analytical variables. In the future, it will be possible to explore how these features influence or regulate the proposed model. Thirdly, data derived exclusively from self-reports by clinical nurses are susceptible to expectancy bias, suggesting the need for complementary, objective data sources in subsequent studies. Despite these constraints, this research provides the first empirical examination of the interplay between hospital ethical climate, moral resilience, and work engagement, which provides new theoretical insights for designing ethical and psychological interventions to enhance nurses’ work engagement and improve their mental health.

## Conclusion

This study demonstrates the mediating role of nurses’ moral resilience in the relationship between hospital ethical climate and work engagement. These findings provide critical insights into the mechanisms linking hospital ethical climate, moral resilience, and work engagement among nurses in China, offering significant implications for clinical nursing management practices. Nursing administrators should prioritize cultivating hospital ethical climate and enhancing moral resilience to improve nurses’ work engagement. Targeted interventions should be developed to strengthen nurses’ perception of the ethical climate and foster their moral resilience by establishing sustainable ethical environments and delivering practical ethics training. Through systematic interventions, these measures will ultimately enhance both nurses’ work engagement and overall quality of care.

## Data Availability

The data that support the findings of this study are available on request from the corresponding author. The data are not publicly available due to privacy or ethical restrictions.
